# An ecological method to understand agricultural standardization in peach orchard ecosystems

**DOI:** 10.1038/srep21675

**Published:** 2016-02-22

**Authors:** Nian-Feng Wan, Ming-Yi Zhang, Jie-Xian Jiang, Xiang-Yun Ji, Hao-Zhang

**Affiliations:** 1 Eco-environment Protection Research Institute, Shanghai Academy of Agricultural Sciences, Shanghai Key Laboratory of Protected Horticultural Technology, Shanghai 201403, China,; 2 Ministry of Education Key Laboratory for Biodiversity Science and Ecological Engineering, Institute of Biodiversity Science, Fudan University, Shanghai 200438, China

## Abstract

While the worldwide standardization of agricultural production has been advocated and recommended, relatively little research has focused on the ecological significance of such a shift. The ecological concerns stemming from the standardization of agricultural production may require new methodology. In this study, we concentrated on how ecological two-sidedness and ecological processes affect the standardization of agricultural production which was divided into three phrases (pre-, mid- and post-production), considering both the positive and negative effects of agricultural processes. We constructed evaluation indicator systems for the pre-, mid- and post-production phases and here we presented a Standardization of Green Production Index (SGPI) based on the Full Permutation Polygon Synthetic Indicator (FPPSI) method which we used to assess the superiority of three methods of standardized production for peaches. The values of SGPI for pre-, mid- and post-production were 0.121 (Level IV, “Excellent” standard), 0.379 (Level III, “Good” standard), and 0.769 × 10^−2^ (Level IV, “Excellent” standard), respectively. Here we aimed to explore the integrated application of ecological two-sidedness and ecological process in agricultural production. Our results are of use to decision-makers and ecologists focusing on eco-agriculture and those farmers who hope to implement standardized agricultural production practices.

Agricultural standardization is based on principles such as simplification, unification, coordination, optimization, etc. on the basis of science, technology and practical experience. The aim of agricultural standardization is to guide and specify agricultural planting, processing, management and sale activities, achieve improved crop yield and quality as well as to promote economic, social and ecological profits^1^,^2^. With the development of economic globalization and trade freedom, people have gradually realized that the standardization of agricultural production is an inevitable trend of modern agricultural development, even in the face of the technical trade barriers constantly strengthened by trade federations and the increasingly worldwide concerns about produce quality and safety.

Since the 1970s, global agriculture standardization has progressed rapidly, especially in some developed countries, where the pre-, mid- and post-production of agriculture have almost realized standardization of methods, and have also formed a more complete production-support system. Reportedly, the standardization levels of agricultural production in the United States, Japan, Germany, and France, to name a few, have ranked among the highest in the world. Agricultural standardization production was seen as a guarantee for increasing farmers’ income, agricultural effectiveness, and rural development^1^,^3^.

At present, the standardization of agricultural production for “green” (environmentally friendly and sustainable) food is in demand in China, because sustainable agriculture has three key differences compared to conventional agriculture: 1) an emphasis on ecology and the environment, 2) quality control of the whole process (pre-production, mid-production and post-production), and 3) traditional techniques that are combined with modern advanced technologies, with each of the production links integrated organically. Thus, the standardization of agricultural production for sustainable agriculture is one of the most important ecological issues for sustainable development of agro-ecosystems and agriculture itself. While ecology has proven to be one of the most effective tools of applied science^4^,^5^,^6^, able to solve many problems^7^,^8^, it has not yet addressed the topic of agricultural standardization.

Certainly, agriculture is closely related to ecology^9^,^10^,^11^,^12^, and agro-ecosystems are based on dynamic processes of material recycling and energy conversion^13^,^14^, whose structure and function, as well as flows (information, material, energy and value flows) vary in the phases of agricultural production. The artificial disturbance to an agro-ecosystem by adopting standardized production methods can also be regarded as a dynamic ecosystem, with three phases (pre-, mid- and post-production).

Agro-ecosystems consist of social, economic and natural factors with different properties that not only have their own respective structure, function and evolution laws, but also interact and restrict one other^15^,^16^. In the process of standardized production, the disturbed agro-ecosystem retains dynamic recycling of materials, energy flow and information exchange, despite some reshaping of its structure and function by the disturbance. However, no matter which type of disturbances standardized production practices impose on a complex agro-ecosystem (through flows of information, material, energy or values), such practices lead to both negative and positive effects on the agro-ecosystem. These negative and positive effects are the intrinsic two-sided attributes that arise after a standardized system for agricultural production disturbed by management practices^17^,^18^,^19^,^20^,^21^,^22^.

China has the largest area of peach cultivation and the largest output of peach yield in the world, with an annual peach cultivation area of 4.520 × 10^5^ ha and an annual peach yield of 4.600 × 10^9^ kg. Standardization of agricultural production in orchards was introduced into China in the 2000s, and then was gradually applied nationwide. In recent years, the standardization of agricultural production has been considered one of the best methods of promoting sustainable agriculture in orchards. This model has been gradually accepted by a large number of farmers and extensively applied in orchards in China.

Here we present a research framework for the standardization of agricultural production based on ecological processes and the principle of ecological two-sidedness, establish the assessment indicator systems for the sustainable production of pre-, mid- and post- production standardization, and propose an eco-index (Standardization of Green Production Index [SGPI]) combined with the Full Permutation Polygon Synthetic Indicator (FPPSI) method, so as to provide a theoretical and practical basis for worldwide agricultural standardization production.

## Results

### Pre-standardization production

Before standardized production was implemented, safe heavy metal levels in soils were seriously exceeded, field irrigation water quality was hardly ideal, and the demands of sustainable food production could not be met. Through adopting physical and biological control techniques of pest management and using ground cover vegetation to improve soil nutrients, the input of agrochemicals was reduced, to some extent the soil environment quality was restored and the indicator levels of heavy metals in soils were all under maximum threshold values, although the content of mercury was close to the threshold value (Table 1). This indicated that curbing the content of mercury in soils was the key to pre-production standardization.

Irrigation water quality was sharply improved through installing floating vegetations in the rivers and constructing sewerage filtration ponds around the peach orchards, and the observed levels were all under critical values. By adopting healthy culture techniques, seedling quality was significantly improved, and the indicator values of excellent rates of bud seedling quality and seedling growth of peach trees increased 15.0% and 8.0%, respectively.

Implementation of standardized production had a definite effect on peoples’ living conditions, as the rates of people with access to sanitary drinking water, garbage disposal, and the sanitary discharge rate of industrial pollutants all reached 100.0%. The centralized sewage treatment rate reached 90.0%, and peoples’ satisfaction rate for environment conditions reached 99.9%.

Analytically, the pre-production index values of negative and positive effects were 0.113 and 0.935, respectively. Thus, the pre-production standardization green index value was 0.121 which indicated that the total conditions of pre-production standardization were suitable for sustainable agriculture as it reached level IV (“Excellent” standard).

### Mid-standardization production

Pests in peach trees were well controlled by adopting physical and biological techniques, and this provided a theoretical and practical basis for pesticide reduction. In addition to the use of ground cover vegetation to improve soil fertility, an inorganic-organic integrated fertilization mode was widely applied to prevent nutrient loss and pollution. The product amounts and the active ingredient weights of the bio-pesticides used were 29.000 kg.hm^−2^ and 12.970 kg.hm^−2^, respectively (crystal lime sulfur accounted for 83.3% of all bio-pesticides), while the product weights and active ingredient weights of conventional chemical pesticides were 12.010 kg.hm^−2^ and 5.000 kg.hm^−2^, respectively, values that were reduced by 50.6% and 40.2%, respectively, through agricultural standardization. The input amount of organic fertilizers was 1.500 × 10^4 ^kg.hm^−2^, while the product weight and the weight of available nutrient in the chemical fertilizers employed were 2.250 × 10^4^ kg.hm^−2^ and 975.000 kg.hm^−2^, respectively, which were reductions of 40.0% and 39.5%, respectively.

Drainage channels and ditches in peach orchards were constructed with the specified standard design, and standard fruit-thinning and bagging techniques were employed. Use of these practices was beneficial to the yield and quality of the peaches as well as cost-saving. With the substantial reduction of chemicals and the standardization of farm operation systems, certain costs were decreased, arthropod diversity was significantly increased, the numbers of neutral groups and natural enemy groups were both substantially increased, and percentage of farmers’ acceptance of standardization production reached 93.8%. However, the input of high-efficiency, environmentally friendly new techniques increased the production cost to some extent, although these techniques provided benefits to related industries.

The mid-production index values of negative and positive effects were 0.341 and 0.900, respectively. Thus, the mid-production standardization green index value was 0.379 which indicated that the implementation of standardization production techniques in this phase was beneficial to peach orchard ecosystems as it reached level III (“Good” standard).

### Post-standardization production

The physical environment of pre-production standardization and the techniques of mid-production standardization are both important influences on post-production indicator values of consumer perception of produce quality, physiochemical characteristics of produce, and fruit sanitary requirements. After standardization production techniques were adopted, the consumer perception of fruit quality had indicator values of perfect for fruit shape, color and luster, neatness of the fruit surface and fruit flavor rate (scores of 90.0, 90.0, 95.0, and 95.0%, respectively), scores that increased by 15.0, 16.0, 18.0, and 15.0% over values in non-standard production schemes. Soluble solids and titratable acids were the most important factors affecting the flavor and quality of fruit, and soluble content and malic acid content were 13.1 and 0.26%, respectively, being increases of 1.8 and 0.06%, respectively. Individual fruit weight (average 0.212 kg) increased by 7.8%, which was a key indicator of enhanced yield and increased income for the farmers. As for sanitary indicators of peach fruits, the contents of heavy metals and chemical pesticide residues were far below maximum established allowable limits, and this guaranteed the safe quality of the produce.

The post-production index values of negative and positive effects were 0.634 × 10^−2^ and 0.824, respectively, so the post-production standardization green index value was 0.769 × 10^−2^, reaching level IV (“Excellent” standard), which indicated that the standardization of pre- and mid-production promoted the healthy development of post-production standardization.

## Discussion

While previous studies have reported on the agricultural practices involved in standardized crop production, ecological methods have not, until now, been applied to this practice. In this study we proposed an integrated methodology of ecological process and ecological two-sidedness from the macro system combined with social, economic and natural factors as well as the crops and environment. To do so, we have divided the existing agricultural process into pre-, mid- and post-production, which allowed us to optimize techniques within a given part of the process.

Before implementing standardized production, farmers used to bag fruits with newspapers, neglected proper thinning of fruit load, and misused agricultural chemicals. As a result, the quality of peach fruits did not meet the requirements of sustainable agriculture. In the standard production scheme studied here, use of chemical pesticides was reduced by adopting biological and physical control techniques. Fertilizer nutrient loss was prevented and fertilizer efficiency was also greatly strengthened by adopting techniques of ground cover vegetation, accurate quantitative fertilization and organic-inorganic integration fertilization modes. In addition, the yield and quality of peaches were raised by standardizing the techniques of fruit thinning and bagging.

To apply ecological principles to both problem analysis and solution, we determined the key factors limiting the development of the peach industry in the study area, and then integrated these techniques with the requirements of sustainable agriculture. The peach yield in the study areas reached 1.628 × 10^4^ kg·hm^−1^, increasing by 9.5%, and the peaches grown there passed National Grade *A* Level of Green Food Certification issued by the China Green Food Development Center. After standardized production systems were implemented, new researchable questions arose: how the complex peach orchard ecosystems responded, how best to objectively evaluate disturbed agro-ecosystems, and how to construct methodological assessments.

The application of ecological principles to agricultural production has been widely done^13^,^23^,^24^, while the ecological two-sidedness concept is just now an emerging focus^17^,^18^,^19^,^20^,^21^,^22^,^25^. However, the integration of both these ecological methodologies has not yet been reported, and their integrated application here is novel.

Certainly, complexity is the intrinsic attribute of any ecosystems^16^,^26^, and an agro-ecosystem disturbed by use of standardized production methods for sustainable food production is no exception. We believe that disturbed agro-ecosystems may have other attributes besides two-sidedness and ecological processes. The SGPI proposed here was combined with Full Permutation Polygon Synthetic Indicator method, and can also be combined with other mathematical theories or methods. In this study, SGPI was an exploratory index to evaluate the two-sidedness involved in process, and was also a quantitative and qualitative index to analyze the negative and positive effects of all disturbance processes. In addition, it will provide a basis for ecologists to dialectically analyze ecological problems by using of complex and two-sidedness thought, and will be beneficial for decision-makers to make scientific decisions as well as for farmers to conduct scientific planting.

## Methods

### Experimented materials

“*Xinfeng*” honey peach (mid-late maturing varieties), 77% cuprousoxide WP, 2% ningnanmycin AS, 72% agricultural streptomycin SP, 45% crystal lime sulphur, 1.2% matrine EC, 50% procymidone WP, 10% difenoconazole WDG, 75% chlorthalonil WP, 65% Zineb WP, 70% thiophanate-methyl WP, 10% imidacloprid WP, 25% chlorbenzuron SC, 2.5% beta-cypermethrin EC, 20% diflubenzuron SC, urea (N 40%), compound fertilizer (N 15%, P_2_O_5_ 15%, K_2_O 15%), organic fertilizer, yellow paper bagging with single layer, solar insecticidal lamp (12v 6w), yellow peach moth sex pheromone, oriental fruit moth sex pheromone, peach leaf miner sex pheromone, plastic tube for suspending sex pheromone, sunflower seed, white clover, irrigation water, and pesticide sprayers.

### Experimental design and method

The study was conducted from 2009 to 2011 in the town of Xinchang, Pudong district of Shanghai Municipality in eastern China (121.41°E, 31.03°N, 4.3 m elevation), where 70.0% of Shanghai peach orchards are concentrated, of which 70.0% conform to standardized agricultural production practices, a higher rate than any other district in Shanghai. For this study, farmers were trained in standardized techniques of agricultural farming under the guidance of scientific researchers in study areas with three replicates (each replicate plot was about 2 ha). Solar insecticidal lamps were installed in each 2-ha areas, and were automatically turned on at 6:00 p.m. and turned off at 5:00 a.m. All lamps were hung 2.0 m above the ground. Clusters composed of one yellow peach moth (*Dichocrocis punctiferalis* Cuenée), one oriental fruit moth (*Grapholitha molesta*), and one peach leaf miner (*Lyonetia clerkella* L.) sex pheromone traps (with the active ingredient in a capillary vessel 10.0 cm long and 1.5 mm in diameter) were hung at 20.0 m intervals in peach orchards, and the attractants were replaced monthly. To enhance the diversity and stability of arthropods and to better control pests in peach orchards, ground cover vegetation (*Trifolium repens* L.) was planted in the peach orchards in the aisles between tree rows.

Thirty peach tree canopies were sampled in a checkerboard pattern in each replicated plot to calculate the diversity and stability of the arthropod community. Each tree, similar to one another in height and vigor, was designated a permanent sampling point to monitor arthropod population dynamics. On each sampling date, each tree was sampled from four directions (east, south, west and north) and at three levels (upper, middle and lower), thereby splitting each tree canopy into 12 resource units^27^,^28^,^29^. The branch beating method was adopted to collect arthropods from the peach tree canopies as per Simon *et al.*^30^. At each canopy level, we spent approximately 3–5 min collecting arthropods and counting the number of individuals of each species of the arthropod communities. We also sampled and measured from one representative twig (20–30 cm long) from each zone in each tree. The species and number of all arthropod taxa were systematically scouted at about 20–30 d intervals. If a species was not well known, it was transferred to 80.0% alcohol with a serial number and later identified in the laboratory.

### Determination of evaluation indicators

**Figure 1:**
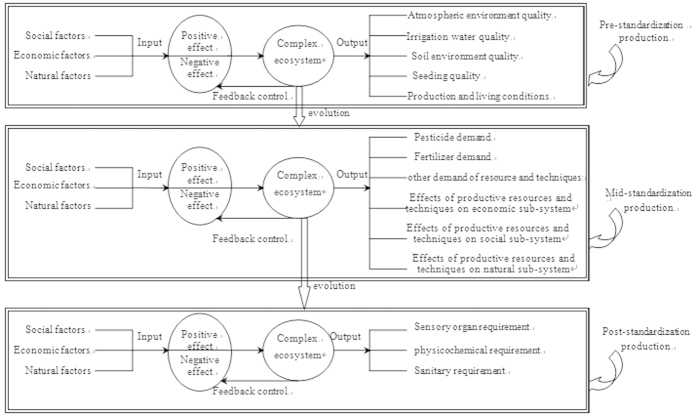
The framework of understanding the standardization of agricultural production in peach orchard ecosystems.

The framework of understanding the standardization of agricultural production in complex ecosystems is shown in Fig. 1. According to standardization theory, standardized production for peaches was divided into pre-, mid- and post-production, and the evaluation indicators for the three components were established respectively. In light of the positive and negative effects on all social, economic and natural elements in a complex agro-ecosystem, the evaluation indicators were divided into positive and negative effects, and the critical value criteria were confirmed with reference to the Shanghai and Nationwide Green Food Industry Standards. Factors of pre-standardization production mainly included seedling quality, water, soil and air quality in the peach production base^31^,^32^,^33^,^34^, as well as the effects on production and living conditions (Table 1). In the mid- production standardization component, agricultural production resources (pesticides, fertilizers, paper bags etc.) and economic cost inputs should be curbed, and attention should be paid to the effects of all management practices on the natural and social subsystems. In lieu of pesticide inputs, physical and biological control techniques should be substituted as much as possible, and any pesticides used should conform to the National Pesticide Use Rule for Green Food^33^. Also, the interval between pesticide applications should conform to recommended safe intervals and the amount of pesticide used should conform to label restrictions. Restrictions on fertilizer use include: (1) use of organic fertilizers whenever possible, (2) limitations on rates of inorganic nutrients (N, P and K), and (3) adherence to the Fertilizer Use Rule for Green Food^32^. Restrictions on cultivation include (1) limitation of drainage channel depths to 0.20–0.30 m, (2) fruit thinning around May 15th, (3) fruit loads of 280 to 350 fruit per tree, and (4) fruit bagging period in mid-to-late May, with a yellow paper bag. Economic costs were mainly composed of costs of pesticides, fertilizer, paper bags, instrument depreciation, labor and other non-chemical controls. The effect of the input of productive materials and techniques on the natural subsystem in the studied peach orchards was measured by considering arthropod diversity, the composition of the three sub-communities of arthropods, their community interrelationships, and the total system’s self-regulatory ability after pest outbreaks. The standard production scheme for peaches requires some inputs of agricultural materials, and within limits, higher use of pesticides, fertilizers, or paper bags increases the economic yield of the farm enterprise. A questionnaire survey was used to determine the degree of farmers’ acceptance of these standardization production techniques. Concrete indicators are shown in Table 2. The indicators of post-production standard procedures mainly involve aspects of sensory, physicochemical, and sanitary requirements^35^. Concrete indicators are shown in Table 3.

Determination of evaluation index: In accordance with the two-sided effect of all factors on complex ecosystems composed of social, economic and natural elements, all indicators were divided into negative and positive influences. Negative factors with extreme low values or positive factors with high values were those that most strongly affected the stability and development of the agro-ecosystems. To quantify the relationship between the two major categories of indicators, we propose the Standardization Green Production Index (*SGPI*) matrix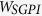, defined as the index optimization matrix of negative indicators divided by the index optimization matrix of positive indicators. The lower the *SGPI*, the more beneficial it would be to the agro-ecosystems. According to typical index classification method, the *SGPI* was divided into four levels (Table 4).

### Evaluation theory and methods

The Full Permutation Polygon Synthetic Indicator (FPPSI) method used here has been applied to optimization of other complex systems^36^,^37^. The standardization process for the value of positive indicators can be described:


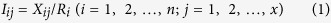


where *I*_*ij*_ was the standardized value of the *i*th indicator to the *j*th project for positive effect evaluation, *X*_*ij*_ was the actual value of the *i*th indicator to the *j*th project for positive effect evaluation, and *R*_*ij*_ was the reference value of the *i*th indicator. Similarly, the standardization process for the value of negative indicators can be described:


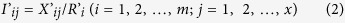


The analytic method for *I*_*ij*_ can be described as follows: (1) the *n* indicators are axes that take a normalized value of 1.0 for each indicator, (2) from these axes, an *n*-sided polygon can be drawn up, the values of all indicators for different projects being scaled over the *n*-sided axes, and (3) the new *n*-sided polygon will be obtained through linking the points of each projection. Then, the area of the new polygon divided by that of a regular *n*-sided polygon with the radius of 1.0 value, will determine the comprehensive index for positive effect indicators:


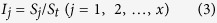


where *I*_*j*_ is the comprehensive index for positive effect indicators, *S*_*j*_ is the area of polygon for the *j*^th^ project, and *S*_*t*_ is the area of regular *n*-sided polygon with the radius of 1.0 value. Similarly, comprehensive index *I’*_*j*_ for negative effect indicators is presented:


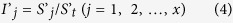


Thus, the matrix *W*_*SGPI*_ for standardization green production evaluation to *x* projects is presented:


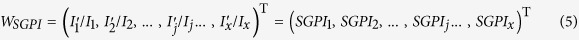


Finally, a reasonable strategy was determined according to the values of *SGPI*, in which it was supposed that the lower the value of *SGPI*, the more reasonable the candidate technique is.

## Additional Information

**How to cite this article**: Wan, N.-F. *et al.* An ecological method to understand agricultural standardization in peach orchard ecosystems. *Sci. Rep.* **6**, 21675; doi: 10.1038/srep21675 (2016).

## Figures and Tables

**Table 1:** The indicators used to evaluate pre-standardization green production in peach orchard ecosystems.

Pre-production standardization green indicator		Indicator type	Unit	Referencevalue ofindicator	Actualvalue ofindicator	Standardizedvalue ofindicator
Atmospheric environment quality	Average daily total suspended particulates	Negative effect	mg·m^−3^	0.300	0.115	0.383
	Average daily SO_2_	Negative effect	mg·m^−3^	0.150	0.043	0.287
	Average daily NO_X_	Negative effect	mg·m^−3^	0.100	0.039	0.390
	Average daily fluoride	Negative effect	μg·dm^−2^·d^−1^	1.800	0.015	0.833 × 10^−2^
Irrigation water quality	Total mercury	Negative effect	μg·L^−1^	1.000	0.220	0.220
	Total cadmium	Negative effect	μg·L^−1^	1.000	0.000	0.000
	Total arsenic	Negative effect	μg·L^−1^	50.000	2.818	5.636 × 10^−2^
	Total lead	Negative effect	μg·L^−1^	100.000	3.283	3.283 × 10^−2^
	Total hexavalent chromium	Negative effect	μg·L^−1^	100.000	7.553	7.553 × 10^−2^
	Total fluoride	Negative effect	mg·L^−1^	2.000	0.295	0.148
Soil environment quality	Total cadmium	Negative effect	mg·kg^−1^	0.300	0.160	0.533
	Total mercury	Negative effect	mg·kg^−1^	0.250	0.247	0.988
	Total arsenic	Negative effect	mg·kg^−1^	25.000	6.158	0.246
	Total lead	Negative effect	mg·kg^−1^	50.000	28.926	0.579
	Total hexavalent chromium	Negative effect	mg·kg^−1^	120.000	57.572	0.480
	Total copper	Negative effect	mg·kg^−1^	100.000	33.480	0.335
Seedling quality	Excellent rate of bud seedling quality	Positive effect	%	100.000	90.000	0.900
	Excellent rate of growth seedling quality	Positive effect	%	100.000	98.000	0.980
The effects on production and living conditions	The qualified rate to reach the drinking water sanitary standards	Positive effect	%	100.000	100.000	1.000
	Sewage centralized treatment rate	Positive effect	%	100.000	90.000	0.900
	Garbage disposal rate	Positive effect	%	100.000	100.000	1.000
	Standard discharge rate of industrial pollution sources	Positive effect	%	100.000	100.000	1.000
	People’s satisfaction rate for environment conditions	Positive effect	%	100.000	99.000	0.990

Note: the value of pH was 7.325 in irrigation water, and the value of pH was 5.394 in soil; BHC and DDT residues were not detected; the total cadmium detection limit was 0.500μg·L^−1^; excellent rate of bud seedling quality: the percentage of well-developed root system, seedling diameters above 6.000 × 10^−3^ m, wound healing well without cracks after grafting, substantial and flush buds without harm, quarantining pests or mechanical injury; excellent rate of growth seedling quality: the percentage of growth seedlings with lateral root number more than 4, length more than 0.150 m and height more than 0.800 m.

**Table 2:** The indicators used to evaluate mid-standardization green production in peach orchard ecosystems.

Mid-production standardization green indicator		Indicator type	Unit	Reference value of indicator	Actual value of indicator	Standardized value of indicator
Pesticide demand	Pesticide input compliance rate for green food	Positive effect	%	100.000	100.000	1.000
	Pesticide compliance rate of safe interval period	Positive effect	%	100.000	100.000	1.000
	Active ingredients of imidacloprid	Negative effect	g·hm^−2^	45.000	21.450	0.477
	Active ingredients of beta-cypermethrin	Negative effect	g·hm^−2^	15.000	12.450	0.830
	Active ingredients of chlorbenzuron	Negative effect	g·hm^−2^	187.500	124.950	0.666
	Active ingredients of diflubenzuron	Negative effect	g·hm^−2^	150.000	60.000	0.400
	Active ingredients of procymidone	Negative effect	g·hm^−2^	375.000	187.500	0.500
	Active ingredients of difenoconazole	Negative effect	g·hm^−2^	75.000	49.950	0.666
	Active ingredients of chlorthalonil	Negative effect	g·hm^−2^	1.688 × 10^3^	703.125	0.417
	Active ingredients of Zineb	Negative effect	g·hm^−2^	1.463 × 10^3^	0.975 × 10^3^	0.667
	Active ingredients of thiophanate-methyl	Negative effect	g·hm^−2^	1.050 × 10^3^	0.525 × 10^3^	0.500
Fertilizer demand	Fertilizer input compliance rate for green food	Positive effect	%	100.000	100.000	1.000
	The input amount of active nitrogen	Negative effect	kg.hm^−2^	937.500	525.000	0.560
	The input amount of active phosphorus	Negative effect	kg.hm^−2^	337.500	225.000	0.667
	The input amount of active potassium	Negative effect	kg.hm^−2^	337.500	225.000	0.667
Other demands of productive resources and techniques	Depth compliance rate of drainage channel and ditch	Positive effect	%	100.000	100.000	1.000
	Compliance rate of fruit-thinning time and fruit-reserving density	Positive effect	%	100.000	100.000	1.000
	Compliance rate of bagging	Positive effect	%	100.000	100.000	1.000
	Coverage rate of yellow bags with single layer	Positive effect	%	100.000	100.000	1.000
	Application rate of physical control	Positive effect	%	100.000	100.000	1.000
	Application rate of biological control	Positive effect	%	100.000	100.000	1.000
Effects of productive resources and techniques on economic sub-system	Pesticide cost	Negative effect	RMB·hm^−2^	3.690 × 10^3^	1.754 × 10^3^	0.475
	Fertilizer cost	Negative effect	RMB·hm^−2^	1.920 × 10^4^	1.320 × 10^4^	0.688
	Instrument depreciation cost	Negative effect	RMB·hm^−2^	120.000	60.000	0.500
	Bagging cost	Negative effect	RMB·hm^−2^	3.750 × 10^3^	1.725 × 10^3^	0.460
	Labor cost	Negative effect	RMB·hm^−2^	1.1250 × 10^4^	9.150 × 10^3^	0.813
	Physical control cost	Negative effect	RMB·hm^−2^·a^−1^	250.000	177.000	0.708
	Biological control cost	Negative effect	RMB·hm^−2^·a^−1^	250.000	120.000	0.480
Effects of productive resources and techniques on natural sub-system	Diversity index of arthropod	Positive effect	%	4.500	4.310	0.958
	percentage of the number of neutral arthropods to the one of pests	Positive effect	%	50.000	49.000	0.980
	percentage of the number of natural enemies to the one of pests	Positive effect	%	70.000	63.000	0.900
Effects of productive resources and techniques on social sub-system	Bio-pesticides promoting the prosperity of pesticide industries	Positive effect	kg.hm^−2^	35.000	29.000	0.829
	Chemical pesticides promoting the prosperity of pesticide industries	Positive effect	kg.hm^−2^	15.000	12.010	0.801
	Organic fertilizers promoting fertilizer industries	Positive effect	kg.hm^−2^	1.500 × 10^4^	1.500 × 10^4^	1.000
	Chemical fertilizers promoting fertilizer industries	Positive effect	kg.hm^−2^	3.000 × 10^3^	2.250 × 10^3^	0.750
	Paper bags promoting bag industries	Positive effect	individual·hm^−2^	1.500 × 10^5^	1.350 × 10^5^	0.900
	Percentage of farmers’ acceptance to standardization production	Positive effect	%	100.000	93.750	0.938

**Table 3:** The indicators used to evaluate post-standardization green production in peach orchard ecosystems.

Post- production standardization green indicator		Indicator type	Unit	Referencevalue ofindicator	Actualvalue ofindicator	Standardizedvalue ofindicator
Sensory organ requirement	Perfective rate of fruit shape	Positive effect	%	100.000	90.000	0.900
	Perfective rate of color and luster	Positive effect	%	100.000	90.000	0.900
	Perfective neatness rate of fruit surface	Positive effect	%	100.000	95.000	0.950
	Perfective flavor rate	Positive effect	%	100.000	95.000	0.950
physicochemical requirement	Percentage of the soluble solids	Positive effect	%	14.000	13.100	0.936
	Percentage of titrable acids (malic acid)	Positive effect	%	0.300	0.260	0.867
	Fruit weight	Positive effect	g	250.000	212.300	0.849
Sanitary requirement	Total arsenic	Negative effect	mg·kg^−1^	0.500	0.010	0.020
	Total lead	Negative effect	mg·kg^−1^	0.200	0.040	0.200
	Total chromium	Negative effect	mg·kg^−1^	0.500	0.050	0.100
	Total cadmium	Negative effect	mg·kg^−1^	0.050	0.010	0.200
	Total fluorin	Negative effect	mg·kg^−1^	0.500	0.100	0.200
	Total mercury	Negative effect	mg·kg^−1^	0.010	0.050 × 10^−2^	0.050
	Imidacloprid	Negative effect	mg·kg^−1^	1.000	0.010	0.010
	Beta-cypermethrin	Negative effect	mg·kg^−1^	0.200	0.200 × 10^−2^	0.010
	Chlorbenzuron	Negative effect	mg·kg^−1^	2.000	0.010	0.005
	Diflubenzuron	Negative effect	mg·kg^−1^	2.000	0.010	0.005
	Procymidone	Negative effect	mg·kg^−1^	2.000	0.200 × 10^−2^	0.001
	Difenoconazole	Negative effect	mg·kg^−1^	0.500	0.010	0.020
	Chlorthalonil	Negative effect	mg·kg^−1^	0.500	0.100 × 10^−2^	0.002
	Zineb	Negative effect	mg·kg^−1^	0.100	0.100 × 10^−2^	0.010
	Thiophanate-methyl	Negative effect	mg·kg^−1^	2.000	0.020	0.010

Note: the perfective rate of fruit shape was determined by the percentage of round and smooth tips of fruits with shallow seams, rounded fruit shapes with no malformation; perfective rate of color and luster was determined by the percentage of staining extent more than one fourth of the desired staining areas when this variety was mature; perfective neatness rate of fruit surface was determined by the percentage of clean peach fruits without pests or mechanical damages; perfective flavor rate was determined by the percentage of peach fruits without unusual smell but with special flavor representative of this variety.

**Table 4:** Classification criterion for the standardization of green production in peach orchard ecosystems.

Level	Value of *SGPI*	Qualitative evaluation
I	>0.750	Bad
II	0.500–0.750	Moderate
III	0.250–0.500	Good
IV	<0.250	Excellent
